# The Tumor Microenvironment of Hepatocellular Carcinoma: Untying an Intricate Immunological Network

**DOI:** 10.3390/cancers14246151

**Published:** 2022-12-13

**Authors:** Camilla Volponi, Aurora Gazzillo, Eduardo Bonavita

**Affiliations:** 1 Laboratory of Cellular and Molecular Oncoimmunology, IRCCS Humanitas Research Hospital, Via Alessandro Manzoni 56, 20089 Rozzano, MI, Italy; 2 Department of Biomedical Sciences, Humanitas University, Via Rita Levi Montalcini 4, 20072 Pieve Emanuele, MI, Italy

**Keywords:** inflammation, tumor immunology, hepatocellular carcinoma, tumor microenvironment, immunotherapy

## Abstract

**Simple Summary:**

In this review, we analyze the contribution of the most abundant immune cell populations in hepatocellular carcinoma (HCC) progression and response to therapy. Moreover, we describe the inflammatory processes associated with the development and evolution of liver cancer. Finally, we provide an updated view on the most recent therapeutical approaches, focusing on the ongoing clinical trials, in which innovative immunotherapies are being tested.

**Abstract:**

HCC, the most prevalent form of primary liver cancer, is prototypically an inflammation-driven cancer developing after years of inflammatory insults. Consequently, the hepatic microenvironment is a site of complex immunological activities. Moreover, the tolerogenic nature of the liver can act as a barrier to anti-tumor immunity, fostering cancer progression and resistance to immunotherapies based on immune checkpoint inhibitors (ICB). In addition to being a site of primary carcinogenesis, many cancer types have high tropism for the liver, and patients diagnosed with liver metastasis have a dismal prognosis. Therefore, understanding the immunological networks characterizing the tumor microenvironment (TME) of HCC will deepen our understanding of liver immunity, and it will underpin the dominant mechanisms controlling both spontaneous and therapy-induced anti-tumor immune responses. Herein, we discuss the contributions of the cellular and molecular components of the liver immune contexture during HCC onset and progression by underscoring how the balance between antagonistic immune responses can recast the properties of the TME and the response to ICB.

## 1. Introduction

The liver is a central organ performing vital functions related to metabolism, detoxification, digestion, and immunity. Because of its anatomy, the liver is continuously exposed to antigens of different origins. The portal vein drains blood from the intestine, spleen, and pancreas to the liver, carrying many foreign molecules derived from both food and the intestinal microbiota. The hepatic immune compartment is thus characterized by a delicate balance between tolerance toward harmless antigens and activation against potential pathogens. Tolerance in the liver is mediated by several non-parenchymal cells which comprise specialized antigen-presenting cells (APCs), such as dendritic cells (DCs), Kupffer cells (KCs), and other non-immune cells, such as liver sinusoidal endothelial cells (LSECs) and hepatic stellate cells [1]. These cells act in concert and maintain a tolerogenic environment through the secretion of immunosuppressive factors, such as transforming growth factor (TGF)-β and interleukin (IL)-10, or via the expression of surface inhibitory ligands, such as programmed death (PD)-ligand-1 (PD-L1). However, this equilibrium can be perturbed by several factors, such as environmental or genetic conditions. Viral infections, metabolic syndrome, alcohol consumption, toxins, and genetic predispositions can lead to the excessive activation of the hepatic immune cells. The consequent inflammatory environment is deleterious for liver functions, and it is associated with an increased insurgence of cancer [2].

Alcohol-related liver disease (ALD) and non-alcoholic fatty liver disease (NAFLD) are becoming the most frequent cause of chronic liver disorders. Notably, over the last ten years, both conditions have contributed to the occurrence of cirrhosis and HCC worldwide and have gradually overcome viral hepatitis infections as the leading cause of liver pathology [3,4]. Among the diverse factors contributing to these temporal trend shifts, modifications in the epidemiology of viral hepatitis, alcohol consumption, and a general increase in obesity and type 2 diabetes are the most common. Remarkably, these conditions share the ability to induce oxidative stress and hepatocellular death, which ultimately lead to exacerbated inflammatory conditions and disease progression. Chronic inflammation represents an essential trigger of carcinogenesis. However, in about 25% of the cases, NAFLD-related HCC can develop in absence of cirrhosis, unlike conditions such as viral infections or ALD [3,4]. Although a plethora of factors can influence liver diseases (e.g., genetic predisposition, lifestyle, and food habits), further fundamental and clinical studies are necessary to understand how different hepatic disorders affect cancer development and progression.

Primary liver cancer is the sixth most frequently diagnosed cancer and the third leading cause of cancer-related death worldwide. Among all the liver cancer types, HCC has the highest prevalence (75–85%) and mortality rate [5]. Unfortunately, most HCC patients are not eligible for curative treatment approaches, such as surgery or liver transplantation, as they often present an advanced disease at the time of diagnosis. The combination of the monoclonal antibodies bevacizumab and atezolizumab, targeting vascular endothelial growth factor (VEGF)-A and PD-L1, respectively, has become the standard of treatment for advanced HCC settings [6]. However, the response rate is still limited to 30% of patients, with a bias towards HCCs that are secondary to viral infections [7,8,9]. Indeed, the benefit of ICB therapy in non-viral related HCCs, such as those associated with non-alcoholic steatohepatitis (NASH), is still questionable [7]. Moreover, the safety of ICB drugs in liver cancer patients remains uncertain due to the high chance of immune-related hepatotoxicity and comorbidities associated with hepatic dysfunction [8,9].

A superior understanding of the immune landscape of the liver is essential for improving the efficacy and safety of immunotherapies based on ICB [10]. In addition to being a site of primary carcinogenesis, many cancer types have a high tropism for the liver, including colorectal, breast, kidney, lung, bladder, and melanoma. Additionally, cancer patients who develop liver metastasis have a dismal prognosis [11]. Mounting evidence suggests that cancer cells which invade or grow in the liver can promote immune tolerance by suppressing both local and systemic immunity [11]. Notably, recent studies exploring the role of secondary liver lesions in response to ICB demonstrated the limited efficacy of immunotherapy due to systemic immune suppression in patients with liver metastasis [11]. Therefore, better knowledge of the mechanisms of the immune tolerance and activation of the liver in both physiological and pathological conditions is necessary to increase response rates and decrease adverse events in liver cancer treatment. Herein, we aim to explore the hepatic immune milieu of HCC by describing the function of each cellular compartment and its mechanisms of activation/suppression. We will discuss the evolution of the immune responses during HCC onset in situ and systemically, revealing the contribution of the tumor immune microenvironment.

## 2. The Role of Inflammation in HCC Occurrence and Progression

Inflammation is a protective mechanism triggered by a damaging event that involves the recruitment of leukocytes, the production of soluble mediators, the remodeling of the extracellular matrix, and the activation of the complement system. From an evolutionary perspective, inflammation was positively selected due to its critical role in host defense against pathogens, tissue repair, regeneration, and, in general, tissue homeostasis. Tumors are inflamed tissues in which different types of inflammation can have opposing functions during the initiation and progression phases [12]. Inflammation as a result of the activation of the immune system against cancerous cells can facilitate elimination before tumor initiation through a process named immunosurveillance [13]. Conversely, the excessive and long-lasting activation of the immune responses, as in the case of chronic inflammatory diseases, can promote tumor outgrowth. Inflammatory signals enhance proliferation, immune cell recruitment, and polarization toward pro-tumorigenic phenotypes. In more advanced disease settings, specific tumor-cell-intrinsic properties can directly modulate antitumor immunity, thus favoring immune escape, cancer progression, and resistance to therapy [14]. In addition, inflammation influences the metastasis process in a wide range of aspects, from cell plasticity to migration and the awakening from a dormant metastatic seed [15]. Clinical evidence has shown that the inhibition of inflammation by non-steroidal anti-inflammatory drugs reduces the risk of cancer-related death in the long term [16].

The persistent immune pressure that actively eliminates the most immunogenic neoplastic cells can also enable the survival of cancer cells, which have acquired features to escape immune control. The result of this process is referred to as immunoediting and consists of the capacity of certain cancer cells to withstand anti-tumor immunity through the loss of tumor antigens, reduced sensitivity to immune effector mechanisms, or via the induction of an immunosuppressive and tolerogenic TME [13].

In a healthy liver, a tolerogenic environment is critical for maintaining homeostasis and preventing liver disease. Abnormal inflammatory conditions can alter the liver’s tolerance. Chronic infections (i.e., HCV and HBV infections), the release of damage-associated molecules (DAMPs) due to toxic liver damage (i.e., alcoholic steatohepatitis—ASH), or liver hereditary diseases (i.e., hemochromatosis) and fat accumulation (i.e., NASH) can disrupt the immune equilibrium of the liver. Moreover, these conditions contribute to the increased rate of death of the hepatocytes, causing an enhanced production of inflammatory cytokines and DAMPs with a consequent influx of activated immune cells, thereby compromising the physiological tolerance [17]. Chronic necroinflammation, consisting of continuous cellular death, compensatory regeneration, and the activation of non-parenchymal cells, is often associated with fibrosis. Proliferation causes replicative stress, DNA damage, and genetic instability, thus supporting the recruitment of immune cells such as the macrophages and neutrophils, which produce reactive oxygen and nitrogen species (ROS and RNS) and favor the accumulation of mutations. The cellular stress, combined with epigenetic modifications, mitochondrial alterations, and senescence, can lead to cancer [17]. Furthermore, inflammation-associated molecules can trigger the de-differentiation of post-mitotic epithelial cells into stem-like cells that have the potential to create tumors [18]. This complex combination of factors might explain the huge intra- and inter-tumor heterogeneity existing among different types of HCC [19]. About 15–20% of all cancers are secondary to a condition of chronic inflammation in the tissue of origin [20]. This frequency is increased to 90% in the case of HCC, which is often diagnosed after a condition of fibrosis and/or cirrhosis [21]. During cell death, hepatocytes release alarmins, such as IL-1α and high mobility group box 1 (HMGB1). These molecules induce the production of inflammatory mediators, such as IL-6, that promote the survival and proliferation of the transformed hepatocytes [22,23]. Both innate and adaptive immune cells are involved in this process. Macrophages and DCs can increase their number by local proliferation, differentiation, or the recruitment of precursors from the circulation, together with monocytes, neutrophils, and innate lymphoid cells (ILCs) [15]. In mouse models of NASH, the antibody-mediated depletion of CD8+ T cells abolishes liver damage, suggesting that the activation of cytotoxic lymphocytes (CTLs) is one of the main causes of hepatocyte death [24]. Another report demonstrated that the depletion of CD8+ T cells and the inhibition of lymphotoxin-B receptor significantly delay the development of tumors in mice affected by chronic liver injuries [25]. Furthermore, certain subsets of CD4+ T cells have shown pro-tumorigenic functions. For example, systemic IL-17A from Th17 cells induced neutrophil infiltration in the adipose tissue, worsening NASH through the release of fatty acids, as well as DNA damage in the hepatocytes and HCC [26]. However, during cancer progression, these cell subsets assume a critical anti-tumorigenic function. Large numbers of tumor-infiltrating CD8+ T cells are correlated with increased overall survival, long-term relapse-free survival, and slower tumor progression [25,27]. Additionally, in a murine model of NASH and HCC, the depletion of CD4+ T cells promoted tumor growth [28]. Innate immune cells carry out a central task in tumor development. In HCC, tumor-associated macrophages (TAMs) can produce cytokines to sustain tumor growth (i.e., IL-1β, TNF, IL-6), promote neo-angiogenesis via the VEGF pathway, and induce cytokine-mediated immunosuppression (i.e., IL 10, TGF-β). The heightened infiltration of TAMs and higher IL-1β serum levels have been associated with a poor prognosis in HCC patients with necrotic tumors. In this work, the authors showed that IL-1β induced the epithelial-to-mesenchymal transition of cancer cells through the hypoxia-inducible factor 1α (HIF-1α), thereby initiating the metastatic process [29]. Moreover, Kuang et al. showed that in the peritumoral stroma of HCC, there is a fraction of monocytes/macrophages which express PD-L1 and mediate the inhibition of the anti-tumoral T cell response [30]. Furthermore, cancer cells can produce factors such as granulocyte-macrophage colony stimulation factor (GM-CSF), VEGF, and poly-unsaturated fatty acids that can attract myeloid-derived suppressor cells (MDSCs). This phenotype of myeloid cells consists of the presence of immature neutrophils and monocytes mobilized from the bone marrow with an increased ability to produce ROS, RNS, prostaglandin E2 (PGE2), and anti-inflammatory cytokines, both systemically and in the TME. These characteristics confer the ability to suppress adaptive immunity and can facilitate tumor progression and metastasis [31]. MDSCs can then be pathologically activated in situ by different pro-inflammatory cytokines and DAMPs, such as interferon (IFN)γ [32], IL-1β [33], IL-6 [34], tumor necrosis factor (TNF)α [35], and HMGB1 [36]. Hypoxia plays an important role in the maintenance of MDSCs through the HIF-1α-mediated expression of ectonucleoside triphosphate diphosphohydrolase 2 in cancer cells [37]. In general, hypoxia has been associated with the shift toward an immunosuppressive TME [38]. In HCC, an increased number of MDSCs correlates with disease progression and reduced overall survival [39]. Many works have also linked liver inflammation, HCC initiation, and progression to the deregulation of complement system activity. The physiological functions of the complement system can enable the recovery from acute liver injury; however, the excessive and long-lasting activation of the complement cascade induces hyperproliferation and tumorigenesis, activating, for example, NF-kB or STAT3 in the KCs and hepatocytes [40]. Notably, unrestrained complement activation has been linked with tumor progression in other cancer settings [41]. In the following paragraphs, we aim to discuss the physiological regulatory nature of the liver immune milieu and the mechanisms through which tolerance can be interrupted, paving the way for HCC.

## 3. Liver Immune Privilege

The basal immune status of the liver is anti-inflammatory and immunotolerant. However, different stimuli can induce a robust immune response. These are the two requirements that find their balance in the physiology of the liver. One of the first pieces of evidence of liver immune privilege identified was the fact that the transplantation of allogenic liver was better tolerated than that of other organs and required lower levels of immunosuppression [42]. The tolerance is mediated by different subsets of resident immune cells that populate the liver: KCs, DCs, natural killer (NK) cells, NK T cells (NKT), CD4+ T cells, CD8+ T cells, unconventional δγ T cells, and B cells [10]. Most of these subsets are involved in the maintenance of liver homeostasis through the suppression of inflammation and immune activation [43]. One of the most relevant effects of immune stimulation is the cytotoxic action of CD8+ T lymphocytes. In the liver, CTLs can be activated, for example, toward virally infected hepatocytes (i.e., HBV or HCV). To prevent liver cell death and organ failure, the activation of CD8+ T cells in the liver is usually transient and suboptimal due to the poor activation of CD4+ T cells, which favors CD8+ T exhaustion and early death [44]. In the case of chronic HCV infections, the lack of CD4+ T cells correlates with an ineffective CD8+ T cell response [45]. These cells gain an exhausted phenotype characterized by T cell immunoglobulin, mucin-domain-containing protein 3, and PD-1 expression [46], in addition to the inability to express effector cytokines [47]. In the liver, DCs also assume a regulatory phenotype [48,49]. Hepatic plasmacytoid dendritic cells (pDCs) produce IL-27 that acts in an autocrine fashion, activating the signal transducer and activator of the transcription 3 signaling pathway, resulting in PD-L1 expression. The exposure of PD-L1 on pDCs induces the differentiation of naïve CD4+ T cells into T regulatory cells (Tregs) [50]. Likewise, liver myeloid DCs express PD-L1 [51] and the enzyme indoleamine 2,3-dioxygenase (IDO), two features that enable Treg expansion, probably in response to the initiation of an inflammatory response. The production of the inflammatory cytokine IFNγ by CTLs triggers IDO transcription and production, inducing, as a negative feedback mechanism, the Treg-mediated suppression of T cell activation [52]. The hepatic sinusoid resident macrophages, known as KCs, also have different immunomodulatory features. These cells express PD-L1, especially upon sensing IL-10. Moreover, they can directly produce IL-10 and TGF-β. The integration of these signals is understood to limit liver tissue injury. KCs can respond to apoptotic cell fragments through TGF-β and IL-10, which promotes the expression of PD-L1 with the consequent suppression of the inflammation [53]. In addition, IFNγ acts on KCs, inducing the expression of IDO [54]. The immunoregulation is also enhanced by the activity of cyclo-oxygenase-2 in activated KCs, which generates the immunosuppressive PGE2 [55]. Moreover, MDSCs and other non-immune cells also contribute to the dampening of immune activation. For instance, liver sinusoidal epithelial cells (LSECs) can promote the inactivation of CD8+ T cells and lead CD4+ T cells to differentiate into Tregs [56], while hepatic stellate cells (HSCs) mediate T cell suppression via PD-L1 expression [57]. HSCs were also shown to synergize with DCs and TGF-β to stimulate Treg differentiation and to skew human monocytes toward an MDSC phenotype [58]. To summarize, both the lymphoid and myeloid compartments, together with non-immune cells, collaborate to generate the tolerant microenvironment characteristic of the liver.

## 4. The Immune Compartment

### 4.1. Myeloid Cells

#### 4.1.1. Dendritic Cells

**Figure 1:**
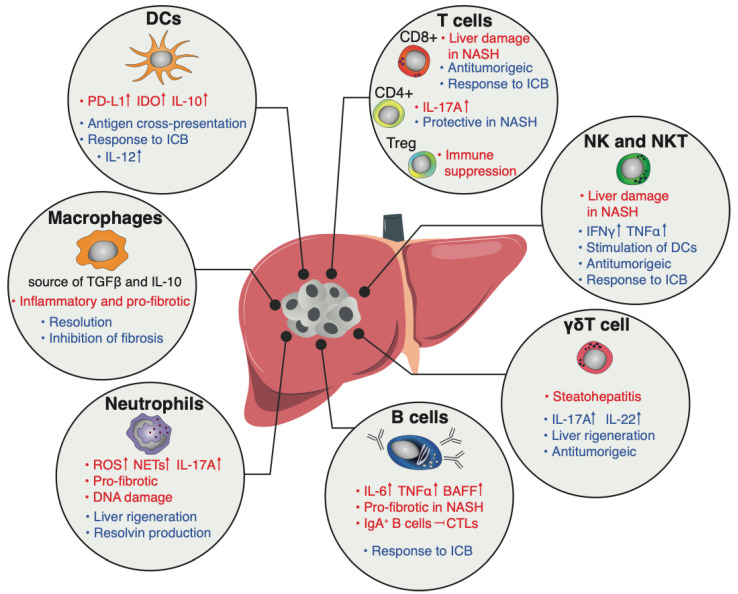
The immune landscape of the HCC microenvironment. A schematic illustration depicting the tumor-promoting (red) and tumor-inhibitory (blue) functions of the most abundant tumor-infiltrating leukocytes.

DCs represent the prototype of APCs. Type I and II conventional dendritic cells (cDC1 and cDC2) are specialized in the priming of CD8+ and CD4+ T cells, respectively. DCs play a central role in the maintenance of the balance between immunoregulation and activation (Figure 1). Hepatic DCs are poorly immunogenic and have a reduced capacity for antigen uptake and processing [48,49,59,60]. Moreover, they express high levels of PD-L1 [51] and represent a major source of IDO and IL-10, which sustain the Treg phenotype [52]. Because of the continuous exposure to intestinal-microbiota-derived LPS, the engagement of Toll-like receptor 4 (TLR4) on liver DCs induces IL-10 and IL-27 production as an alternative to pro-inflammatory cytokines [61]. DC anergy can be further fostered by the inhibitory molecules expressed by T cells. For example, CTLA-4, on resting T cells, can bind to CD80 and CD86 on DCs, inducing IDO production [62]. Additionally, pDCs contribute to the reduction in liver immune activation. These cells express high levels of nucleotide-binding oligomerization domain 2 protein, which mediates the inhibition of the TLR4 and 9 signaling pathways and the augmentation of PD-L1 expression [63]. However, similar to other liver-resident leukocytes, DCs can adapt their phenotype and become inflammatory. For instance, the lipid content of liver DCs is correlated with their capacity to trigger or inhibit the immune response. DCs with low lipid levels are more prone to active T cells, NK cells, and NK T cells, whereas high-lipid-content DCs have a reduced antigen-processing capacity and induce Treg differentiation [64,65]. Yet, DCs are involved in the pathogenesis of different types of liver disease, such as fibrosis, NAFLD/NASH, and HCC. For example, cDC1s have been shown to promote inflammatory T cell reprogramming in NASH mouse models and to expand in the blood and liver of NAFLD/NASH patients [66]. Notably, however, cDC1s were found to be critical for the priming of antigen-specific CD8+ T cells, and they have emerged as essential for the control of high-immunogenic HCCs [67]. In a recent report, Zhang and colleagues, using an integrated single-cell RNA sequencing approach, identified three intra-tumoral clusters of DCs in HCC patients. In this dataset, cDC1s were characterized by the expression of *CLEC9A*, *XCR1,* and *CADM*. In contrast, cDC2s specifically expressed *CD1C*, *FCER1A*, and *CLEC10A*. A non-classical LAMP3+ DC subset was also identified. This cluster expressed high levels of *CCR7*, *LAMP3*, *CD80*, and *CCL19* [68]. Interestingly, the gene expression profile of LAMP3+ DCs underlines their migratory capacities and potential interactions with effector, exhausted, and regulatory T cells [69,70]. Remarkably, this DC population is highly conserved across mouse and human tumors, albeit that their functional role has not been fully characterized [71]. Besides conventional DCs, monocyte-derived DCs (moDCs) also play an important role in sustaining inflammation during pathological settings. In the liver, moDCs have been shown to promote the progression of steatohepatitis [72,73]. However, whether DCs enhance or antagonize liver inflammation and pathologies is not completely clear. Pradere et al. have demonstrated that DCs are dispensable in the development of liver fibrosis [74], although Blois et al. identified DCs as negative regulators of liver fibrotic angiogenesis [75]. In HCC, DCs have been shown to increase the uptake of extracellular lipids, particularly by accumulating oxidatively truncated lipids, which can alter their functionality by inhibiting the cross-presentation [76,77]. Moreover, DCs are also known to express chemokines, such as CCL22, which are able to recruit Tregs in the TME [78], while semi-mature DCs enhance HCC progression by inducing an immunoregulatory phenotype of the B cells [79].

#### 4.1.2. Macrophages and Monocytes

Liver macrophages are found in the lumen of the liver sinusoids, where they mediate the response to pathogens, the regulation of hepatocyte metabolism, and the maintenance of tolerance [80,81] (Figure 1). They comprise tissue-resident KCs that, in physiological conditions, represent the major cellular macrophage fraction in mice, as well as monocyte-derived macrophages originating from circulating monocytes [82]. There is also evidence of a particular subset of monocyte-derived macrophages that reside in the capsular zone, which addresses potential pathogens arriving from the peritoneum [83]. KCs originate from embryonic precursors that are seeded before birth, and they self-renew through local proliferation [82,84,85,86]. Conversely, monocyte-derived macrophages differentiate from the circulating monocytes recruited into the liver upon inflammatory signals, such as CCL2 [87,88]. Indeed, during NAFLD and NASH, KC mortality increases due to impaired self-renewal, and their niche is repopulated by monocyte-derived macrophages [89]. In both mice and humans, newly recruited macrophages can either differentiate into short-lived macrophages or into self-renewing, long-lived resident macrophages, which resemble KCs [90,91]. However, these macrophages are often less mature and more inflammatory than KCs, causing fibrosis and liver injury [92,93]. There is also another group of macrophages that differentiate from incoming monocytes, known as lipid-associated macrophages. Generally, these are considered pro-inflammatory, although a recent study conducted by Daemen et al. demonstrated that a subpopulation of CCR2-dependent lipid-associated macrophages display a protective and anti-fibrogenic role [94]. Single-cell RNA sequencing studies recently shed a light on the different subsets of macrophages that are found in mouse and human liver in both healthy and pathological conditions [84,95,96]. During NAFLD, NASH, or cirrhosis, the number of recruited monocyte-derived macrophages increases, while resident KCs are depleted. Unsurprisingly, macrophages have been largely studied for their implication in inflammatory liver disorders. One of the first pieces of evidence was shown in obese mice and rats, where macrophage depletion prevented the development of steatosis and insulin resistance [97]. However, not all macrophage subsets contribute to liver pathogenesis in equal ways. Transcriptomic analysis of the macrophages in mice affected by obesity or steatosis revealed that inflammatory markers are exclusively expressed by monocyte-derived macrophages and not by KCs [98]. These data have been corroborated by further analyses of the macrophages during NASH [89,94,99]. Some subpopulations of macrophages can also modulate hepatic insulin sensitivity through the production of IL1β and insulin-like growth-factor-binding protein 7 (IGFB7) [100,101]. Conversely, a protective function of the anti-inflammatory macrophages was recently described. Their immunoregulatory function mediated the death of HSCs during NAFLD, as well as the production of extracellular-matrix-degrading factors, which helped to prevent fibrosis [102,103].

#### 4.1.3. Neutrophils

Neutrophils are bone-marrow-derived granulocytes that are found in the circulation and recruited in tissues upon inflammation. They exhibit anti-microbial and cytotoxic functions mediated by phagocytosis and the release of ROS, cytokines, and neutrophil extracellular traps (NETs) [104]. The accumulation of neutrophils as a consequence of inflammation is also observed in the context of the liver. Patients with ALD have an increased number of neutrophils in both the peripheral blood and liver, which is correlated with poor clinical outcomes [105,106]. Moreover, in these patients, the neutrophils are more activated and prone to degranulation and NET formation, and they produce higher levels of ROS [107,108] (Figure 1). Even though a higher neutrophil–leukocyte ratio (NLR) has been correlated with NASH and fibrosis severity [109], patients with NASH are more susceptible to infections [110]. The pathological role of the neutrophils is exerted by their granular proteins (i.e., neutrophil elastase, lipocalin 2, and lactoferrin) [111], ROS, enzymes (i.e., myeloperoxidase) [112], and NETs. Moreover, neutrophil-derived IL-17A constitutes a relevant pro-fibrotic stimulus known to activate HSCs to produce collagen and TGF-β [113,114]. In turn, the activated HSCs increase the expression of GM-CSF to recruit more neutrophils, thereby initiating a positive feedback loop [114]. However, these newly recalled neutrophils are impaired in their anti-microbial functions. Notably, patients with cirrhosis are likely to undergo acute-on-chronic liver failure (ACLF), characterized by microbial infections associated with neutrophil dysfunction [115]. In HCC, tumor-associated neutrophils (TANs) are involved in cancer progression. ROS can cause DNA damage that, consequently, induces oncogene mutations and tumor development [116]. In addition, released factors such as NETs trigger the metastatic process, in addition to remodeling the extracellular matrix through metalloprotease activation [117]. Furthermore, peritumoral neutrophils represent a source of hepatocyte growth factor (HGF) and IL-17A, which promote cell division and cancer progression [117,118]. Neutrophils are also capable of chemoattracting Tregs and macrophages, thus generating an immunosuppressive TME. Moreover, neutrophils can produce high amounts of arginase-1, which depletes L-arginine, thereby impairing the proliferation and activation of T cells [119,120]. A recent study conducted by Leslie et al. corroborated this concept, showing that the inhibition of neutrophil recruitment in NASH-associated HCC led to a profound remodeling of the TME and that this was associated with a stronger response to immunotherapy [121]. However, neutrophils can also mediate liver regeneration. Neutrophils can indeed support the acquisition of a reparative phenotype by the macrophages during acute liver injury [122]. They can release metalloproteases that tackle fibrosis [123] and participate in the synthesis of lipid mediators, such as lipoxins, which are involved in the resolution of inflammation [124].

### 4.2. Lymphoid Cells

#### 4.2.1. T Cells

Hepatic T lymphocytes need to be perfectly orchestrated in order to maintain the equilibrium between immune tolerance and response. CD8+ effector T cells are the main mediators of the specific cytotoxic response to infected or transformed cells. The effects of the cytotoxicity range from the killing of the infected cell to the release of damage-associated molecular patterns (DAMPs) and cytokines that drive inflammation (Figure 1). Hence, the excessive activation of T cell-mediated immunity could result in substantial hepatocyte death with consequent liver failure. On the other hand, the incapacity of T cells to act against infected cells or pathogens originating from the circulation leads to the spreading of organ and systemic disease conditions. The T cell compartment is a complex group of different cell types that act in concert to ensure liver homeostasis. Naïve CD8+ and CD4+ T cells (T_N_) need to be primed by cells capable of presenting the cognate antigen (Ag) on MHC class I or class II, respectively. This first signal, together with the ones mediated by costimulatory molecules and cytokines, guides the differentiation and activation of T_N_ into effector and memory T cells [125]. Usually, the enrolled APCs are DCs and macrophages, while non-immune cells, such as the epithelial cells of non-lymphoid organs, cannot come into contact with T cells. In the liver, the structure of the sinusoidal vessels, characterized by the absence of tight junctions between endothelial cells and by the lack of a coherent basal membrane (fenestrated capillaries), allows the hepatocytes to present Ags to the CD8+ T cells [126,127]. Additionally, LSECs can take up soluble gut-derived Ags, thereby favoring tolerance [128]. However, a study by Klein and Crispe showed that hepatocytes were sufficient in generating an effective CD8+ T cell response in an orthotopic liver transplantation model [129]. Currently, it has been demonstrated that hepatocytes can induce the local activation and proliferation of T cells [130]. The fate of these primed cells consists of either death (apoptosis or emperipolesis) [131,132] or differentiation into functional or dysfunctional CD8+ T effector cells [133]. Transcriptional analysis experiments showed that the dysfunction of CD8+ T cells did not necessarily correlate with an exhausted phenotype but was characterized, instead, by a gene expression program involved in the tissue remodeling process. This transcriptional state can be reversed by the administration of IL-2 [133], whose physiological production is probably ascribable to Ag-specific CD4+ T cells. When CD8+ T cells reach their effector phenotype, they are arrested on the LSECs in a platelet-dependent manner and crawl along the sinusoids. Subsequently, they generate protrusions that lengthen through the endothelial fenestrae to establish the immunological synapse with the hepatocytes, which results in the killing of the infected or transformed cells [127]. Interestingly, the reduction in or occlusion of intercellular spaces, as a consequence of liver fibrosis, inhibits the recognition and elimination of infected or cancerous cells, permitting the chronicity of viral infections and the development of HCC, respectively [127]. The completion of the immune response is promoted by the liver constitutive production of IL-15, which induces the development of resident memory CD8+ T cells upon CTL activation. These cells remain in the organ after the conclusion of the inflammatory process, ready to respond in the case of a secondary infection [134].

#### 4.2.2. B Cells

B cells are known for their ability to become antibody-secreting plasma cells, to present antigens, and to produce cytokines that regulate the T cell-mediated response. In the liver, B cells assume specific functions, and they are involved in the development of NASH and HCC (Figure 1). B cells can be organized into three main cellular subsets: B1, B2, and B regulatory cells (Bregs) [135]. B1 cells originate from the fetal liver and mainly localize in the peritoneal and pleural spaces. They are further subdivided into B1a cells, which produce antibodies against self-antigens, and B1b cells, which can class-switch. Immature B2 cells migrate to the spleen, where they maturate. B2 cells from the marginal zone respond to blood-borne pathogens and lipid antigens, while follicular B2 cells constitute the majority of the peripheral and secondary lymphoid organ B cell population. It has been demonstrated that obesity promotes a B cell response against the “self” [136]. B cells can infiltrate the mesenteric adipose tissue and migrate to the liver, where they promote inflammation [137]. Mounting evidence shows that hepatic B cells are increased in patients with NAFLD and NASH, together with the serum IgGs against oxidative-stress-derived epitopes (OSE). Consistently, the depletion of the B2 subset induces a decrease in the Th1 responses and consequent NASH-related inflammation [138]. Barrow et al. demonstrated that in NASH preclinical models, B cells accumulate in the liver and express cytokines, such as IL-6 and TNFα, inducing inflammation and fibrogenesis [139]. Indeed, these two cytokines are involved in CD4+ T cell activation and differentiation into Th1 cells in NASH and steatosis. As a mediator of activation, the B cell activating factor (BAFF) is gaining increasing relevance, as it has been found to contribute to NASH pathogenesis, acting on the adipose tissue, hepatic tissue, and B cells [139,140]. Additionally, the alteration in gut permeability occurring in NAFLD and NASH can trigger B cell activation, for example, through TLR4 or BCR engagement [139,141]. Regarding the T-cell-activating function of B cells, in NASH patients, B cells were found to gather around regions rich in T cells [138]. B cells may also be involved in the activation of HSCs through TNFα production, which can then differentiate into myofibroblasts secreting collagen and factors that favor the fibrotic condition [142]. Since HCC is a common consequence of NASH, the B cells might be involved in liver carcinogenesis and tumor progression. The number of tumor-infiltrating B cells positively correlates with tumor progression in NASH-driven HCC patients [143]. Specifically, IgA^+^ B cells express PD-L1 and inhibit CTL activation. Moreover, Bregs can dampen anti-tumor immunity and elicit tumor growth by interacting with cancer cells and producing IL-10. However, different works showed that liver B cells may also have an anti-tumoral role and that their depletion enables tumor growth and development [27]. Interestingly, in other tumor types, the B cells are enriched in the responders to ICB compared to non-responders [144]. An explanation for these conflicting results could be that the B cell contribution to NASH insurgence and HCC depends on the stage of the disease, the model studied, the specific B cell subset analyzed, and the microbiota composition [136].

#### 4.2.3. NK Cells and ILCs

K cells represent 40% of the lymphocytes populating the human liver, thus playing a dominant role in the maintenance of the immune equilibrium of the organ (Figure 1). There are two main subtypes of hepatic NK cells: liver-resident NK cells (lrNKs) and conventional NK cells (cNKs) [145,146]. lrNKs belong to the type 1 innate lymphoid cells (ILC1s), which differ from conventional NK cells in their ontogenesis, expressed markers, function, and localization [147]. lrNKs are NK cells that reside in the organ, while cNKs circulate from the liver to the periphery and vice versa. These cells belong to innate immunity, even though they are critical for the generation of an efficient adaptive immune response. NK cells are specialized in the killing of cancerous and infected cells, and this strong cytotoxicity makes allows them to be involved in many liver diseases. cNKs represent the dedicated cytotoxic subset able to produce perforin and granzyme, while ILC1s are non-cytotoxic cells that secrete IFNγ and TNFα and are thus able to generate a cytokine response [148,149,150]. The inflammatory or regulatory phenotype of liver NK cells depends on microenvironmental stimuli, such as KC-derived IL-18 and IL-1β, or signals from apoptotic cells [151,152]. Notably, the genetic blockade of IL-1 receptor 8 (IL-1R8) on NK cells mediated resistance to HCC and CMV infections [153]. Even if the specific roles of the cNKs and ILC1s are yet to be clarified, a transcriptional analysis of these cells showed that cNKs shift toward the expression of cytotoxicity-related genes, while ILC1s shift toward genes involved in the immunoregulation [154]. This could suggest a possible interplay between these two subsets of innate immune cells in the equilibrium between immune activation and suppression, which can be disturbed by disease conditions. For instance, in acute HBV patients, the severity of liver damage correlates with a highly inflammatory phenotype of NK cells, characterized by the increased expression of activating receptors, IFNγ production, and a degranulating capacity [155]. The activated NK cells can also have an immunomodulatory function, as in the case of HCV infection, where they can kill CD4+ T cells and sustain chronic pathology [156]. Conversely, in chronic HBV patients, NK cells lose their effector functions, and this condition may be associated with the persistence of the virus in the liver [157,158]. We have already discussed how the augmentation of the inflammation levels can lead to liver fibrosis and, eventually, HCC. NAFLD is one of the causes of increased liver inflammation, and NK cells have been found to be involved in the progression to NASH, even if their specific role is still debated [159,160]. In HCC patients, the decreased number or functionality of NK cells is associated with a poor prognosis. The activity of both cNKs and lrNKs is impaired in the TME, suggesting that tumor cells are selected to quench the NK-mediated immune activation [161,162]. NK cells in the TME show a decreased expression of activating receptors (i.e., TIGIT, NKG2D, and NKp30) and an upregulation of inhibitory receptors (i.e., NKG2A, TIM3, and CD96) [162,163]. Further analysis of the HCC-infiltrating NK cells revealed their exhausted phenotype and their association with a reduced disease-free and overall survival [162]. Moreover, intratumoral NK cells are characterized by a shifted metabolism and a secretory phenotype that supports tumor growth by releasing TME-modifying factors, such as VEGF, matrix metallopeptidase 9, and angiogenin. These alterations in the NK cell functions in the TME can be triggered by different cell types, such as cancer-associated fibroblasts (CAFs), macrophages, and Tregs [151,164,165,166,167]. Even if NK cells generally have anti-tumoral functions, Sun et al. demonstrated that the lrNK cell number positively correlates with tumor growth, development, and poor tumor outcomes in HCC patients [168]. Since this subset is non-cytotoxic and generally prone to immunoregulation, these results suggest that it may have a pro-tumoral activity.

#### 4.2.4. iNKT

Invariant NKT (iNKT or type I NKT) cells are a group of adaptive immune cells that own a particular semi-invariant TCR that is able to recognize lipids presented by the non-classical class I-like molecule CD1d [169]. iNKT cells have been implicated in anti-tumoral immunity via various mechanisms (Figure 1). For example, they can produce large amounts of IFNγ and stimulate IL-12 secretion by DCs [170], thus activating the NK- and CD8+ T cell-mediated response [171]. Moreover, iNKT cells can directly kill transformed cells through the secretion of perforin and granzyme B [172], as well as through Fas–FasL interaction in vivo [173]. Their protective role was demonstrated in mouse models lacking NKT cells (*Jα18*^−/−^ mice), where the immunosurveillance against methylcholanthrene-induced sarcomas was lost and reconstituted upon wild type liver iNKT cell transfer [174]. Among liver leukocytes, the iNKT cell subset was found to be the most efficient in mediating anti-tumoral immunity [174]. The central role of iNKT cells in the response to cancer is also underlined by the fact that iNKT cells are either depleted or impaired in terms of IFNγ production in solid tumors [175,176]. However, another class of NKT cells, the type II NKT cells, have an immunoregulatory function and have been shown to elicit tumor growth in different mouse models [177,178]. As reviewed by Marrero et al., the dichotomic features of NKT subsets offer different contributions to the development of inflammatory liver diseases [179]. Indeed, type II NKT cells, due to their inhibitory activity, can protect the liver from inflammation and tissue damage, while iNKT cells mediate these effects and are often associated with the chronicity of the diseases [176,180,181,182,183,184]. Wolf et al. demonstrated that the long-term feeding on a choline-deficient high-fat diet mediates NASH and related HCC by promoting the liver infiltration of activated iNKT cells and CD8+ T cells [24]. Conversely, the inhibition of iNKT cells prevented (N)ASH insurgence in preclinical settings [181,185,186].

#### 4.2.5. γδT Cells

The γδT cells represent about 15–25% of the total liver T cells [187]. These T cells are characterized by γδTCR instead of the canonical αβTCR and own a variety of TLRs, pattern recognition receptors (PRRs), and C-type lectin receptors that enable the recognition of a wide spectrum of pathogen-associated molecular patterns (PAMPs) and DAMPs [188,189]. Since their activation is not dependent on the identification of a specific antigen, they can be considered as early responders involved in the beginning of inflammation. In the liver, they have been demonstrated to regulate regeneration via the production of IL-22 and IL-17 [190] (Figure 1). However, the protective role of γδT cells may potentially be independent of IL-17 [191], which has a controversial function, since it is involved, for example, in both the prevention of liver damage and the development of fibrosis and NAFLD in mice [192,193,194,195]. Additionally, γδT cells promote diet-induced steatohepatitis in murine models [196]. Concerning HCC, the γδT cells are subject to a depletion in the TME, in addition to the impairment of their cytotoxicity and IFNγ production [197]. In fact, an increased number of γδT cells is associated with higher survival among HCC patients undergoing surgical resection, thus representing a promising tool for cancer immunotherapy [198].

## 5. Metabolic Syndrome and Liver Cancer

Metabolic syndrome (MetS) is defined as a clinical condition characterized by the concomitant occurrence of at least three metabolic risk factors including obesity, dyslipidemia, impaired glucose metabolism, high blood pressure, and low levels of high-density lipoprotein cholesterol (HDL-c) [199]. Since obesity, insulin resistance/type II diabetes, and hyperlipidemia are also the main causes of NAFLD, the latter can be considered as the hepatic manifestation of MetS [200,201]. NAFLD encompasses the whole spectrum of fatty liver diseases, from simple liver steatosis to steatohepatitis, being unrelated to alcohol abuse. These pathologies are characterized by the accumulation of lipids within the hepatocytes, which can then lead to chronic liver inflammation, fibrosis, cirrhosis, and, eventually, HCC [200,202]. Notably, patients with MetS are likely to have high levels of liver fat [203]. One of the factors most significantly influencing the insurgence of steatosis is insulin resistance. This metabolic condition induces an augmentation of hepatic de novo lipogenesis and adipose tissue lipolysis, with a consequent increase in the number of hepatocyte-free fatty acids (FFAs) that are accumulated as triglycerides, as well as the production of dysregulated numbers of adipokines and inflammatory cytokines (i.e., TNFα, IL-6, and IL-1β) [204]. Yet, microbiota variations due to a diet rich in fats and fructose can induce an increase in bowel permeability and fatty acid production, promoting obesity and FFA entrance into the liver [205]. These factors, combined with individual genetic and epigenetic predispositions, can induce hepatic inflammation, hepatocyte death, HSC activation, and fibrosis [200]. FFAs in the liver, on one side, can be stored in the form of triglycerides, sustaining NAFLD, while from the other side, they can generate lipotoxicity, which induces mitochondrial dysfunction, ROS production, ER stress, and inflammasome assembly [200,206]. Mitochondrial dysfunction can also be sustained by increased levels of TNFα, which are a result of insulin resistance in the adipose tissue. In turn, the excessive release of ROS enhances the oxidation of low-density lipoprotein (LDL) particles and, together with the latter, the inflammatory and pro-fibrotic activation of KCs and HSCs. Oxidative stress is also closely connected to ER stress [207,208]. Prolonged ER stress can cause unfolded-protein-response (UPR)-related hepatocyte death, with a consequent release of DAMPs, other inflammatory signals, and molecules able to spread the ER stress condition to neighboring cells [209]. Chronic inflammation and lipid accumulation are characteristics of NAFLD/NASH that guide the transition to HCC. In NASH, “ballooning hepatocytes” may be facilitated in becoming cancerous by their altered cytoskeleton, which could prevent apoptosis or cell cycle arrest [210]. Furthermore, alterations in metabolic substrates both sustain cancer cell metabolism and remodel the hepatic immune milieu. For example, the increased availability of circulating fatty acids and glucose positively selects those cells that are able to perform glucose and lipid catabolism. Consistently, the shift toward these two metabolic pathways in cancer cells has been demonstrated by their altered proliferation and migration, as well as chemoresistance [211,212]. Moreover, recent work by Liu et al. suggests that increased liver glycogen storage correlates with augmented carcinogenesis [213]. Concerning the reprogramming of the immune microenvironment, it was shown that in a NASH mouse model, lipotoxicity causes the depletion of liver CD4+ T cells and hepatocarcinogenesis [28]. Moreover, in HCC, TAMs are more prone to fatty acid oxidation, a characteristic that promoted tumor cell migration in vitro via IL-1β [29]. Tregs can concomitantly exploit fatty acid oxidation, synthesis, and glycolysis, and this represents a major metabolic advantage over the effector T cells, which mainly rely on glycolysis. Glucose in the TME is limited because of its consumption by many cell types, such as cancer cells, peritumoral monocytes, and other pro-inflammatory cells [210,214,215]. Glucose paucity mainly penalizes CD8+ T cells. Conversely, it favors cells, such as Tregs, which are able to exploit other organic molecules as an alternative [216]. Moreover, in a pancreatic cancer model, it was shown that cancer-cell-derived IL-1β and a high-fat diet could increase the number of pro-tumorigenic lipid-loaded TAMs, enabling tumor progression [217]. Many works have studied the effects of lipid intake and metabolism on the DCs [218,219,220,221]. A high-fat diet dampens DC anti-tumor functions. Herber et al. described how DCs, in tumor-bearing mice, increase the expression of the macrophage scavenger receptor 1 (MSR1), boosting the uptake of exogenous lipids from the plasma and resulting in the loss of their antigen processing and presentation capabilities [64]. Together, the data suggest that the significant amount of liver lipids that distinguishes the NAFLD and NASH conditions from one another could foster the suppressive nature of the hepatic immune milieu, easing the carcinogenesis process.

## 6. Therapy of Liver Cancer

Liver transplantation (LT) and liver resection (LR) represent the desirable treatment approaches for HCC [222]. LT is the most efficacious treatment because, in addition to eradicating the tumor, it removes the underlying chronic liver pathologies that eventually present themselves. Transplantation, however, is subject to organ availability, and long waiting lists expose patients to the risk of tumor progression and loss of surgery eligibility. Therefore, LR constitutes the most common treatment for HCC in the earlier stages [21]. Despite the fact that curative surgeries show a 5-year overall survival (OS) in 70% of the treated patients [4], these interventions must be carried out with some restrictions. Surgery is, indeed, recommended only when tumors display limited dimensions and spread [222]. However, more than 60% of patients are not eligible for such interventions. Moreover, events such as post-hepatectomy liver failure and tumor recurrence are rife [223]. An alternative approach to HCC is interventional radiology, including radiofrequency/microwave ablation (RFA/MWA) and intra-arterial intervention [224]. RFA and MWA consist of the application of localized heat to the inside of the tumor mass, which triggers cancer cell necrosis. These techniques are non-invasive, but their effectiveness is affected by the tumor dimensions and the presence of large blood vessels (heat sink effect) [222,225,226]. Intra-arterial interventions include trans-arterial chemoembolization (TACE) and selective internal radiotherapy (SIRT). Recently, new techniques, such as stereotactic beam radiotherapy (SBRT) and proton beam therapy (PBT), are becoming increasingly common due to their superior precision, even if their performance is still under evaluation [227]. Systemic therapy constitutes the treatment of choice in patients diagnosed with intermediate- or late-stage HCC. The most commonly used chemotherapeutic agents for HCC are multi-kinase inhibitors [21]. These molecules block the activity of pro-angiogenic and pro-proliferative tyrosine kinase (TK) growth factor receptors and/or RAF kinases. Nowadays, there are four MIKs approved for the treatment of HCC: lenvatinib (first/second line), sorafenib (second line), regorafenib (third line), and cabozantinib (third/fourth line). However, these drugs present several contraindications. Despite having demonstrated their safety, MKIs are not specific for tumor cells and their toxicity is particularly elevated. Sorafenib, for example, has shown adverse effects such as skin reactions, diarrhea, asthenia, hypertension, and the worsening of liver functions, leading, in the 10–15% of cases, to the interruption of the treatment [228,229]. Moreover, MKIs have only moderate effectiveness, and resistance is an ordinary phenomenon due to the redundancy of the TK-mediated signaling pathways [230,231]. The identification of biomarkers predicting the responsiveness of patients to MIKs would be critical, yet the only clinically applied biomarker for Ramucirumab (anti-VEGFR2) prescription is based on the alpha-fetoprotein (AFP) serum levels. Notably, in an effort to deliver more tailored therapies to patients, the molecular characterization of the HCC subtypes provided significant insights [19]. Among the most innovative approaches, ICB therapies have displayed promising results and changed HCC clinical treatment [232]. Nivolumab and pembrolizumab (anti-PD1) monotherapies, together with the association of nivolumab and ipilimumab (anti-CTLA-4), have been approved by the Food and Drug Administration (FDA) as a third-line treatment for HCC patients who have progressed on sorafenib [233,234]. In addition, both MKIs and ICB have been associated with anti-angiogenic monoclonal antibodies (anti-VEGF(R) and anti-TGFβ) in normalizing the tumor vessels [6,235]. Currently, ramucirumab is the only anti-angiogenic monoclonal antibody approved as a third-line monotherapy [236], while the combination of bevacizumab (anti-VEGF) and atezolizumab (anti-PDL1) is used as a first-line therapy [6]. Although anti-PD(L)1 monoclonal antibodies showed promising results, their success is restricted to a relatively low percentage of patients, being around 15% in the CheckMate 459 Trial [237]. Therefore, complementary strategies aiming to increase the T cell response are necessary in order to overcome the immunosuppressive nature of the liver microenvironment. Examples of the possible options could be adoptive cell transfer therapy (i.e., the infusion of autologous T cells stimulated or engineered to be reactive against the tumor ex vivo), vaccines with tumor-derived antigens, and oncolytic viruses [10]. Other potential immunotherapies are alternative antibody-based therapies (such as anti-CD105, anti-endosialin, etc.), the administration of immunostimulatory cytokines, mTOR and IDO inhibitors, or senescence-inducing compounds [10,222]. Among the new treatments in ongoing clinical trials, there are also chimeric antigen receptor (CAR) T cells engineered against glypican 3 (GPC3), AFP, and other tumor-specific antigens. Moreover, the use of arginine-depleting enzymes such as pegargiminase, DC-based vaccines, and monoclonal antibodies against immunoregulatory cytokines, such as IL-27, is currently being tested. The ongoing clinical trials based on innovative approaches to treating liver cancer are listed in Table 1.

## 7. Conclusions

Liver TME is an extremely complex site of immunological activities. In homeostatic conditions, the protection of the hepatic tissue is ensured by the maintenance of a tolerogenic milieu. However, the disruption of such a delicate equilibrium inevitably results in liver damage. Inflammation, in different forms, represents the main cause of liver dysfunction and disease. The acute and chronic activation of immune cells, fueled by necroinflammation, ultimately results in tumorigenesis. The contributions of distinct immune cell compartments to HCC development and progression, as well as the ICB response, have been recently addressed. Yet, a wealth of evidence remains contradictory. The large diffusion of single-cell-based approaches, such as single-cell RNA sequencing and spatial transcriptomics, enable the high-resolution characterization of the liver TME, thereby unveiling transcriptionally distinct immune subpopulations potentially playing a role in HCC and possibly leading to the identification of novel targets for immunotherapy.

ICB combinations have become the standard of care for HCC in advanced disease settings, although the portion of responsive patients is still unsatisfying. Indeed, the biggest challenge in the immunotherapy of HCC is the design of new treatments that are simultaneously more effective and less toxic. Emerging innovative approaches include adoptive cell therapy, chimeric antigen receptor T cells, new-generation vaccines, and oncolytic viruses [238,239]. Moreover, combination therapies of ICB with drugs targeting the inflammatory TME on both the cellular and molecular level might dramatically improve patient outcomes [239]. Nevertheless, the refinement of new and innovative ICB combinations will require an additional understanding of liver-specific immunity and greater effort in fundamental basic research. The design of innovative pre-clinical models of HCC that are able to resemble the genetic heterogeneity of human cancer and the cirrhotic/steatotic environments is essential for this purpose. Lastly, crucial problems in tackling liver cancer are early detection and disease monitoring. Therefore, an in-depth characterization of the systemic inflammatory switches associated with tumor progression and/or response to therapy will be critical for patient selection and the establishment of precision immunotherapy as the cornerstone of treatments for HCC.

## Figures and Tables

**Table 1:** List of innovative therapies for liver malignancies.

Category	Clinical Trial Summary	Conditions	Interventions	URL/Identifier Accessed on 10 October 2022
Monoclonal Antibodies	Durvalumab and Tremelimumab in Resectable HCC	Hepatocellular Carcinoma	Drug: Tremelimumab	https://ClinicalTrials.gov/show/NCT05440864
	A Study to Evaluate Tislelizumab Combined With Sitravatinib as Adjuvant Therapy in Participants With HCC at High Risk of Recurrence After Curative Resection	Hepatocellular Carcinoma	Drug: Tislelizumab + Sitravatinib	https://ClinicalTrials.gov/show/NCT05407519
	Tislelizumab in the Systematic Treatment of Advanced Hepatocellular Carcinoma	Hepatocellular Carcinoma	Drug: Tislelizumab	https://ClinicalTrials.gov/show/NCT04996459
	Camrelizumab in Patients With Unresectable Hepatocellular Carcinoma	Unresectable Hepatocellular Carcinoma	Drug: Camrelizumab	https://ClinicalTrials.gov/show/NCT04947956
	A Trial of Hepatic Arterial Infusion Combined With Apatinib and Camrelizumab Versus Apatinib and Camrelizumab for C-Staged Hepatocellular Carcinoma in BCLC Classification	C-staged Hepatocellular Carcinoma in BCLC Classification	Combination Product: Hepatic Arterial Infusion combined with Apatinib and Camrelizumab|Combination Product: Apatinib combined with Camrelizumab	https://ClinicalTrials.gov/show/NCT05313282
	Trial of Atezolizumab and Bevacizumab With SRF388 or Placebo in Patients With Hepatocellular Carcinoma	Hepatocellular Carcinoma	Drug: SRF388|Drug: Atezolizumab|Drug: Bevacizumab|Drug: Placebo	https://ClinicalTrials.gov/show/NCT05359861
	Adjuvant Immunotherapy With Toripalimab Following Curative-Intent Ablation for Recurrent Hepatocarcinoma	Hepatocellular Carcinoma	Drug: Toripalimab|Procedure: Thermal ablation	https://ClinicalTrials.gov/show/NCT05240404
	Camrelizumab Combined With Apatinib Mesylate for Perioperative Treatment of Resectable Hepatocellular Carcinoma	Hepatocellular Carcinoma|Immunotherapy|Molecular Targeted Therapy	Drug: Camrelizumab|Drug: Apatinib Mesylate|Procedure: TACE treatment|Procedure: Radical surgery	https://ClinicalTrials.gov/show/NCT04521153
	Anti-CTLA4-NF mAb (BMS986218), Nivolumab, and Stereotactic Body Radiation Therapy for the Treatment of Metastatic Solid Malignancies	Advanced Lung and Liver cancer	Biological: Anti-CTLA4 Monoclonal Antibody BMS-986218|Biological: Nivolumab|Radiation: Stereotactic Body Radiation Therapy	https://ClinicalTrials.gov/show/NCT04785287
	A Study to Determine Whether Chemotherapy, Bevazicumab, and Atezolizumab is Better Than Chemotherapy Alone in Patients With Advanced Liver Cancer	Combined Hepatocellular Carcinoma and Cholangiocarcinoma|Stage III Liver Cancer|Stage IV Liver Cancer	Biological: Atezolizumab|Biological: Bevacizumab|Procedure: Biospecimen Collection|Drug: Cisplatin|Procedure: Computed Tomography|Procedure: Conventional Magnetic Resonance Imaging|Drug: Gemcitabine Hydrochloride	https://ClinicalTrials.gov/show/NCT05211323
	Feasibility and Efficacy of Perioperative Nivolumab With or Without Relatlimab for Patients With Potentially Resectable Hepatocellular Carcinoma (HCC)	Hepatocellular Carcinoma	Drug: Nivolumab|Drug: Relatlimab	https://ClinicalTrials.gov/show/NCT04658147
	Camrelizumab, Apatinib Plus HAIC Versus Camrelizumab and Apatinib for HCC With Portal Vein Invasion: a Randomized Trial	Hepatocellular Carcinoma	Procedure: FOLFOX-HAIC|Drug: Camrelizumab|Drug: Apatinib	https://ClinicalTrials.gov/show/NCT05198609
	A Phase II Study of Nivolumab + Ipilimumab in Advanced HCC Patients Who Have Progressed on First Line Atezolizumab + Bevacizumab	Advanced Hepatocellular Carcinoma |Unresectable Hepatocellular Carcinoma	Biological: Ipilimumab|Biological: Nivolumab	https://ClinicalTrials.gov/show/NCT05199285
	Futibatinib and Pembrolizumab for the Treatment of Advanced or Metastatic FGF19 Positive BCLC Stage A, B, or C Liver Cancer	Advanced Hepatocellular Carcinoma	Drug: Futibatinib|Biological: Pembrolizumab|Other: Quality-of-Life Assessment	https://ClinicalTrials.gov/show/NCT04828486
	Neoadjuvant Regorafenib Plus Durvalumab (MEDI4736) in Patients With High-Risk Hepatocellular Carcinoma	Stage IB Hepatocellular Carcinoma AJCC v8|Stage II Hepatocellular Carcinoma AJCC v8|Stage IIIA Hepatocellular Carcinoma AJCC v8	Biological: Durvalumab|Drug: Regorafenib	https://ClinicalTrials.gov/show/NCT05194293
	Durvalumab (MEDI4736) and Tremelimumab in Combination With Either Y-90 SIRT or TACE for Intermediate Stage HCC With Pick-the-winner Design	Hepatocellular Carcinoma Non-resectable	Drug: Tremelimumab|Drug: Durvalumab|Procedure: Y-90 SIRT|Procedure: TACE	https://ClinicalTrials.gov/show/NCT04522544
	Atezolizumab in Combination With a Multi-Kinase Inhibitor for the Treatment of Unresectable, Locally Advanced, or Metastatic Liver Cancer	Advanced Hepatocellular Carcinoma|Metastatic Hepatocellular Carcinoma|	Biological: Atezolizumab|Drug: Cabozantinib|Drug: Lenvatinib	https://ClinicalTrials.gov/show/NCT05168163
	HAIC Combined With Camrelizumab and TKI for Unresectable Hepatocellular Carcinoma After TACE Failure	Unresectable Hepatocellular Carcinoma	Drug: Camrelizumab|Drug: HAIC|Drug: TKI	https://ClinicalTrials.gov/show/NCT05135364
	Hyperbaric Oxygen Therapy Combined Camrelizumab in Patients With Advanced/Metastatic Hepatocellular Carcinoma	Combinational Immunotherapy|Hepatocellular Carcinoma Non-Resectable|Hyperbaric Oxygen Therapy	Combination Product: Hyperbaric Oxygen Therapy plus Camrelizumab	https://ClinicalTrials.gov/show/NCT05031949
	Nivolumab and ADI-PEG 20 Before Surgery for the Treatment of Resectable Liver Cancer	Resectable Hepatocellular Carcinoma	Biological: Nivolumab|Biological: Pegargiminase|Procedure: Resection	https://ClinicalTrials.gov/show/NCT04965714
	TSR-022 (Anti-TIM-3 Antibody) and TSR-042 (Anti-PD-1 Antibody) in Patients With Liver Cancer	Adult Primary Liver Cancer|Advanced Adult Primary Liver Cancer|Localized Unresectable Adult Primary Liver Cancer	Drug: TSR-022 and TSR-042	https://ClinicalTrials.gov/show/NCT03680508
	Pembrolizumab With or Without Elbasvir/Grazoprevir and Ribavirin in Treating Patients With Advanced Refractory Liver Cancer	Hepatocellular Carcinoma|Hepatitis C Infection|Refractory Liver Carcinoma	Drug: Elbasvir/Grazoprevir|Other: Laboratory Biomarker Analysis|Biological: Pembrolizumab|Drug: Ribavirin	https://ClinicalTrials.gov/show/NCT02940496
	Nivolumab With or Without Ipilimumab in Treating Patients With Resectable Liver Cancer	Hepatocellular Carcinoma|Resectable Hepatocellular Carcinoma	Biological: Ipilimumab|Biological: Nivolumab	https://ClinicalTrials.gov/show/NCT03222076
	Tremelimumab With Chemoembolization or Ablation for Liver Cancer	Heptocellular Cancer|Biliary Tract Neoplasms|Liver Cancer|Hepatocellular Carcinoma|Biliary Cancer	Drug: Tremelimumab|Procedure: RFA|Procedure: TACE|Procedure: Cryoablation	https://ClinicalTrials.gov/show/NCT01853618
	Lenvatinib Combined Anti-PD1 Antibody for the Advanced Hepatocellular Carcinoma	Hepatocellular Carcinoma|Anti-PD1 Antibody|Liver Diseases	Drug: Lenvatinib|Drug: Opdivo|Drug: Camrelizumab|Drug: Keytruda|Drug: Toripalimab|Drug: Sintilimab|Drug: Tislelizumab	https://ClinicalTrials.gov/show/NCT04627012
	Study of Safety and Tolerability of PDR001 in Combination With Sorafenib and to Identify the Maximum Tolerated Dose and/or Phase 2 Dose for This Combination in Advanced Hepatocellular Patients	Hepatocellular Carcinoma	Drug: PDR001|Drug: Sorafenib	https://ClinicalTrials.gov/show/NCT02988440
Drugs/molecules/particles	Yttrium Y 90 Glass Microspheres, Atezolizumab, and Cabozantinib for the Treatment of Unresectable or Locally Advanced Hepatocellular Carcinoma	Locally Advanced Hepatocellular Carcinoma|Recurrent Hepatocellular Carcinoma|Unresectable Hepatocellular Carcinoma	Biological: Atezolizumab|Procedure: Biopsy|Drug: Cabozantinib S-malate|Radiation: Yttrium Y 90 Glass Microspheres	https://ClinicalTrials.gov/show/NCT05327738
	TheraSphere With and Without Durvalumab and Tremelimumab for HCC	Hepatocellular Carcinoma	Device: TheraSphere Y-90 Glass Microsphere Therapy|Drug: Durvalumab (Imfinzi) Immunotherapy|Drug: Tremelimumab Immunotherapy	https://ClinicalTrials.gov/show/NCT05063565
	GEN2 Directed Cancer Immunotherapy Trial	Hepatocellular Carcinoma|Metastatic Cancer	Drug: GEN2 (HSV-Thymidine Kinase-m2 and hGM-CSF Genes)	https://ClinicalTrials.gov/show/NCT04313868
	IRX-2, Cyclophosphamide, and Nivolumab in Treating Patients With Recurrent or Metastatic and Refractory Liver Cancer	Recurrent Hepatocellular Carcinoma|Refractory Liver Carcinoma	Drug: Cyclophosphamide|Biological: Cytokine-based Biologic Agent IRX-2|Biological: Nivolumab	https://ClinicalTrials.gov/show/NCT03655002
	BO-112 and Pembrolizumab for the Treatment of PD-1/PD-L1 Refractory Liver Cancer	Advanced Hepatocellular Carcinoma |Refractory Hepatocellular Carcinoma	Biological: Nanoplexed Poly I:C BO-112|Biological: Pembrolizumab	https://ClinicalTrials.gov/show/NCT04777708
	NBTXR3, Radiation Therapy, Ipilimumab, and Nivolumab for the Treatment of Lung and/or Liver Metastases From Solid Malignancy	Metastatic Malignant Neoplasm in the Liver|Metastatic Malignant Neoplasm in the Lung	Other: Hafnium Oxide-Containing Nanoparticles NBTXR3|Biological: Ipilimumab|Biological: Nivolumab|Radiation: Radiation Therapy	https://ClinicalTrials.gov/show/NCT05039632
	Study Of OX40 Agonist PF-04518600 Alone And In Combination With 4-1BB Agonist PF-05082566	Neoplasms	Drug: PF-04518600|Drug: PF-04518600 plus PF-05082566	Completed
	Doxorubicin and Interleukin-2 in Treating Patients With Liver Cancer That Cannot Be Removed by Surgery	Liver Cancer	Biological: Aldesleukin|Drug: Doxorubicin Hydrochloride	https://ClinicalTrials.gov/show/NCT00004248
	Study Of OX40 Agonist PF-04518600 Alone And In Combination With 4-1BB Agonist PF-05082566	Neoplasms	Drug: PF-04518600|Drug: PF-04518600 plus PF-05082566	https://ClinicalTrials.gov/show/NCT02315066
	TACE-HAIC Combined With TKIs and Immunotherapy Versus TACE Alone for Hepatocellular Carcinoma With PVTT	Hepatocellular Carcinoma	Procedure: TACE-HAIC|Procedure: TACE|Drug: Targeted Therapy|Drug: PD-1 Inhibitors	https://ClinicalTrials.gov/show/NCT05535998
	Safety and Immune Response to a Multi-component Immune Based Therapy (MKC1106-PP) for Patients With Advanced Cancer	Solid cancers	Biological: PSMA/PRAME	https://ClinicalTrials.gov/show/NCT00423254
Vaccines	Neoantigen Dendritic Cell Vaccine and Nivolumab in HCC and Liver Metastases From CRC	Hepatocellular Carcinoma|Hepatocellular Cancer|Colorectal Cancer|Colorectal Carcinoma|Liver Metastases	Biological: Neoantigen Dendritic Cell Vaccine|Drug: Nivolumab	https://ClinicalTrials.gov/show/NCT04912765
	Vaccine Therapy in Treating Patients With Liver or Lung Metastases From Colorectal Cancer	Colorectal Cancer|Metastatic Cancer	Biological: Falimarev|Biological: Inalimarev|Biological: Sargramostim|Biological: Therapeutic Autologous Dendritic Cells	https://ClinicalTrials.gov/show/NCT00103142
	GP96 Heat Shock Protein-Peptide Complex Vaccine in Treating Patients With Liver Cancer	Liver Cancer	Biological: gp96	https://ClinicalTrials.gov/show/NCT04206254
	DNAJB1-PRKACA Fusion Kinase Peptide Vaccine Combined With Nivolumab and Ipilimumab for Patients With Fibrolamellar Hepatocellular Carcinoma	Fibrolamellar Hepatocellular Carcinoma (FLC)	Drug: DNAJB1-PRKACA Peptide Vaccine|Drug: Nivolumab|Drug: Ipilimumab	https://ClinicalTrials.gov/show/NCT04248569
	Safety and Efficacy Study of Mix Vaccine in Hepatocyte Carcinoma Patient	Liver Neoplasms	Biological: MV|Other: Standard Treatment	https://ClinicalTrials.gov/show/NCT02338778
	Vaccine Therapy in Treating Patients With Advanced or Metastatic Cancer	Breast Cancer|Colorectal Cancer|Gallbladder Cancer|Gastric Cancer|Head and Neck Cancer|Liver Cancer|Ovarian Cancer|Pancreatic Cancer|Testicular Germ Cell Tumor	Biological: TRICOM-CEA(6D)	https://ClinicalTrials.gov/show/NCT00027534
	Vaccine Therapy and Radiation to Liver Metastasis in Patients With CEA-Positive Solid Tumors	Liver Neoplasms	Drug: rV-CEA(6D)/TRICOM-rF-CEA(6D)/TRICOM|Drug: rF-CEA(6D)/TRICOM|Drug: Recombinant Fowlpox-GM-CSF|Drug: Celecoxib	https://ClinicalTrials.gov/show/NCT00081848
	Vaccine Therapy in Treating Patients With Liver or Lung Metastases From Colorectal Cancer	Colorectal Cancer|Metastatic Cancer	Biological: Falimarev|Biological: Inalimarev|Biological: Sargramostim|Biological: Therapeutic Autologous Dendritic Cells	https://ClinicalTrials.gov/show/NCT00103142
	Immunotherapy in Treating Patients With Resected Liver Metastases From Colon Cancer	Colorectal Cancer|Metastatic Cancer	Biological: Carcinoembryonic Antigen RNA-Pulsed DC Cancer Vaccine	https://ClinicalTrials.gov/show/NCT00003433
	Vaccine Therapy With or Without Sirolimus in Treating Patients With NY-ESO-1 Expressing Solid Tumors	Solid neoplasms	Biological: DEC-205/NY-ESO-1 Fusion Protein CDX-1401|Other: Laboratory Biomarker Analysis|Other: Pharmacological Study|Drug: Sirolimus	https://ClinicalTrials.gov/show/NCT01522820
CAR T cells	Anti-CEA CAR-T Cells to Treat Colorectal Liver Metastases	Colorectal Cancer|Metastatic Liver Cancer	Drug: Anti-CEA CAR-T Cells	https://ClinicalTrials.gov/show/NCT05240950
	Interleukin-15 Armored Glypican 3-specific Chimeric Antigen Receptor Expressed in Autologous T Cells for Hepatocellular Carcinoma	Liver Cell Carcinoma	Genetic: CATCH T Cells|Drug: Cytoxan|Drug: Fludara	https://ClinicalTrials.gov/show/NCT05103631
	Interleukin-15 and -21 Armored Glypican-3-specific Chimeric Antigen Receptor Expressed in T Cells for Pediatric Solid Tumors	Liver Cancer|Rhabdomyosarcoma|Malignant Rhabdoid Tumor|Liposarcoma|Wilms Tumor|Yolk Sac Tumor	Genetic: CARE T Cells|Drug: Cytoxan|Drug: Fludara	https://ClinicalTrials.gov/show/NCT04715191
	ECT204 T-Cell Therapy in Adults With Advanced HCC	Hepatocellular Carcinoma|Liver Cancer, Adult|Liver Neoplasm|Metastatic Liver Cancer	Biological: ECT204 T Cells	https://ClinicalTrials.gov/show/NCT04864054
	GPC3 Targeted CAR-T Cell Therapy in Advanced GPC3 Expressing Hepatocellular Carcinoma (HCC)	Hepatocellular Carcinoma|Hepatocellular Cancer|Metastatic Hepatocellular Carcinoma	Drug: Cyclophosphamide|Biological: CAR-T Cells|Drug: Fludarabine	https://ClinicalTrials.gov/show/NCT05003895
	Novel GPC3 CAR-T Cell Therapy for Hepatocellular Carcinoma	Hepatocellular Carcinoma	Biological: GPC3-CAR-T Cells	https://ClinicalTrials.gov/show/NCT05344664
	GPC3-Targeted CAR-T Cell for Treating GPC3 Positive Advanced HCC	Hepatocellular Carcinoma	Biological: CAR-T Cell Immunotherapy	https://ClinicalTrials.gov/show/NCT04121273
	Study of ET140203 T Cells in Adults With Advanced Hepatocellular Carcinoma (ARYA-1)	Hepatocellular Carcinoma|Liver Cancer|Liver Neoplasm|Metastatic Liver Cancer	Biological: ET140203 Autologous T Cell Product	https://ClinicalTrials.gov/show/NCT04502082
	Interleukin-15 Armored Glypican 3-specific Chimeric Antigen Receptor Expressed in T Cells for Pediatric Solid Tumors	Liver Cancer|Rhabdomyosarcoma|Malignant Rhabdoid Tumor|Liposarcoma|Wilms Tumor|Yolk Sac Tumor	Genetic: AGAR T Cells|Drug: Cytoxan|Drug: Fludara	https://ClinicalTrials.gov/show/NCT04377932
	GPC3-CAR-T Cells for Immunotherapy of Cancer With GPC3 Expression	Hepatocellular Carcinoma|Immunotherapy|CAR|GPC3 Gene Inactivation|T Cell|Squamous Cell Lung Cancer	Biological: GPC3 and/or TGFβ Targeting CAR-T Cells	https://ClinicalTrials.gov/show/NCT03198546
	Glypican 3-Specific Chimeric Antigen Receptor Expressed in T Cells for Patients With Pediatric Solid Tumors (GAP)	Liver Cancer	Genetic: GAP T Cells|Drug: Cytoxan|Drug: Fludara	https://ClinicalTrials.gov/show/NCT02932956
	Glypican 3-Specific Chimeric Antigen Receptor Expressing T Cells for Hepatocellular Carcinoma (GLYCAR)	Hepatocellular Carcinoma	Genetic: GLYCAR T Cells|Drug: Cytoxan|Drug: Fludarabine	https://ClinicalTrials.gov/show/NCT02905188
	AFP^c332^T in Advanced HCC	Hepatocellular Cancer|AFP Expressing Tumors	Genetic: Autologous Genetically Modified AFP^c332^T Cells	https://ClinicalTrials.gov/show/NCT03132792
	T Cell Receptor Immunotherapy Targeting NY-ESO-1 for Patients With NY-ESO-1 Expressing Cancer	Melanoma|Meningioma|Breast Cancer|Non-Small-Cell Lung Cancer|Hepatocellular Cancer	Biological: Anti-NY ESO-1 mTCR PBL|Drug: Cyclophosphamide|Drug: Fludarabine|Drug: Aldesleukin	https://ClinicalTrials.gov/show/NCT01967823
	CEA-Expressing Liver Metastases Safety Study of Intrahepatic Infusions of Anti-CEA Designer T Cells	Liver Metastases	Biological: Anti-CEA 2nd-Generation Designer T Cells	https://ClinicalTrials.gov/show/NCT01373047
	CAR-T Hepatic Artery Infusions and Sir-Spheres for Liver Metastases	Liver Metastases	Biological: Anti-CEA CAR-T Cells|Device: Sir-Spheres	https://ClinicalTrials.gov/show/NCT02416466
	CAR-T Hepatic Artery Infusions or Pancreatic Venous Infusions for CEA-Expressing Liver Metastases or Pancreas Cancer	Liver Metastases	Biological: Anti-CEA CAR-T Cells	https://ClinicalTrials.gov/show/NCT02850536
Other Cell-based	Combination of Cryosurgery and NK Immunotherapy for Tumors in Transplanted Liver	Liver Tumor|Evidence of Liver Transplantation	Device: Cryosurgery|Biological: NK Immunotherapy	https://ClinicalTrials.gov/show/NCT02849015
	Combination of Irreversible Electroporation and NK Immunotherapy for Recurrent Liver Cancer	Recurrent Liver Carcinoma	Device: Irreversible Electroporation|Biological: Natural Killer	https://ClinicalTrials.gov/show/NCT03008343
	Safety Study of Liver Natural Killer Cell Therapy for Hepatoma Liver Transplantation	Liver Cirrhosis|Hepatocellular Carcinoma|Evidence of Liver Transplantation	Biological: Liver NK Cell Inoculation	https://ClinicalTrials.gov/show/NCT01147380
	Safety and Efficiency of γδ T Cell Against Liver Cancer	Liver Cancer	Procedure: Cryosurgery or IRE Surgery|Biological: γδ T cell|Other: γδ T cells/ A Cryosurgery or IRE	https://ClinicalTrials.gov/show/NCT03183219
	Safety and Efficiency of γδ T Cell Against Hepatocellular Liver Cancer	Liver Cancer	Biological: DC-CIK Cells|Biological: γδ T Cells|Biological: γδ T/DC-CIK Cells	https://ClinicalTrials.gov/show/NCT02425735
	A Study of DC-CIK Immunotherapy in the Treatment of Solid Tumors	Liver Cancer|Kidney Cancer|Nasopharyngeal Cancer|Lung Cancer|Colorectal Cancer|Breast Cancer	Other: CELL	https://ClinicalTrials.gov/show/NCT04476641
	Safety and Efficacy of “Immuncell-LC” in TACE Therapy	Carcinoma, Hepatocellular	Biological: Immuncell-LC	https://ClinicalTrials.gov/show/NCT02856815
	RFA or Surgical Resection Combined With Neo-MASCT for Primary HCC: a Phase II Trial	Primary Liver Cancer|Radiofrequency Ablation|Immunotherapy|Hepatectomy	Biological: Neo-MASCT	https://ClinicalTrials.gov/show/NCT03067493
	Biological Therapy in Treating Patients With Metastatic Cancer	Solid Neoplasms	Biological: CEA RNA-pulsed DC Cancer Vaccine	https://ClinicalTrials.gov/show/NCT00004604
	Efficacy and Safety of Immuncell-LC Group and Non-Treatment Group in Hepatocellular Carcinoma Patients	Hepatocellular Carcinoma	Biological: Immuncell-LC	https://ClinicalTrials.gov/show/NCT00699816
	CIK Treatment for HCC Patient Underwent Radical Resection	Carcinoma, Hepatocellular	Biological: Cytokine-Induced Killer Cells	https://ClinicalTrials.gov/show/NCT01749865
	Autologous Immune Cell Therapy in Primary Hepatocellular Carcinoma Patients Following Resection and TACE Therapy	Primary Hepatocellular Carcinoma	Biological: DC-TC+GM-CSF	https://ClinicalTrials.gov/show/NCT01828762
	Combination Therapy of Microwave Ablation and Cellular Immunotherapy for Hepatocellular Carcinoma	Hepatocellular Carcinoma	Biological: Adoptive Immunotherapy|Procedure: MWA	https://ClinicalTrials.gov/show/NCT02851784
	Combine TACE and Autologous Tcm Immunotherapy Versus TACE Alone for HCC With MVI After Radical Resection	Hepatocellular Carcinoma|Malignant Neoplasm	Combination Product: TACE plus Autologous Tcm Immunotherapy|Procedure: TACE	https://ClinicalTrials.gov/show/NCT03575806
Oncolytic viruses	Study of Talimogene Laherparepvec With Atezolizumab for Triple Negative Breast Cancer and Colorectal Cancer With Liver Metastases	Metastatic Triple-Negative Breast Cancer|Metastatic Colorectal Cancer	Biological: Talimogene Laherparepvec|Biological: Atezolizumab	https://ClinicalTrials.gov/show/NCT03256344
	Hepatocellular Carcinoma Study Comparing Vaccinia Virus Based Immunotherapy Plus Sorafenib vs. Sorafenib Alone	Hepatocellular Carcinoma (HCC)	Biological: Pexastimogene Devacirepvec (Pexa Vec)|Drug: Sorafenib	https://ClinicalTrials.gov/show/NCT02562755
	Study of Talimogene Laherparepvec With Atezolizumab for Triple Negative Breast Cancer and Colorectal Cancer With Liver Metastases	Metastatic Triple-Negative Breast Cancer|Metastatic Colorectal Cancer	Biological: Talimogene Laherparepvec|Biological: Atezolizumab	https://ClinicalTrials.gov/show/NCT03256344
Alternative approaches	Huaier Granule for Prevention of Recurrence and Metastasis of Hepatocarcinoma After Radical Hepatectomy	Hepatic Carcinoma	Drug: Huaier Granule	https://ClinicalTrials.gov/show/NCT01770431
Prediction/biomarkers	Predicting Response to Systemic Therapies for Hepatocellular Carcinoma (HCC)	Hepatocellular Carcinoma Non-Resectable|Effect of Drug	Diagnostic Test: Radiological Evaluation	https://ClinicalTrials.gov/show/NCT05543304
	Evaluation of Treatment Predictors Reflecting Beta-catenin Activation in Hepatocellular Carcinoma	Hepatocellular Carcinoma Non-resectable	Combination Product: Fluorine-18 Fluorocholine	https://ClinicalTrials.gov/show/NCT04965454
	Immune Cells as a New Biomarker of Response in Patients Treated by Immunotherapy for Advanced Hepatocellular Carcinoma	Hepatocellular Carcinoma	Other: Patients with Hepatocellular Carcinoma	https://ClinicalTrials.gov/show/NCT05044676
	Liquid Biopsy in Hepatocellular Carcinoma	Hepatic Carcinoma Malignant Primary Non-Resectable	Diagnostic Test: Cell-Free DNA	https://ClinicalTrials.gov/show/NCT04111029
	Identification of Image Phenotypes to Predict Recurrence After Resection of Hepatocellular Carcinoma	CT Scans Prior to Surgery With a Least 2 Years of Follow-up	Other: Non-Intervention	https://ClinicalTrials.gov/show/NCT05235490
	Analysis of Expression of Specific Markers and Their Prognostic Significance in Hepatocellular Carcinoma	Hepatocellular Carcinoma	Other: Retrospective Analysis of Already Archived Samples	https://ClinicalTrials.gov/show/NCT00911196
